# Case Report of a pathologically confirmed vascular parkinsonism with early cognitive impairment and Behavioral disturbance

**DOI:** 10.1186/s12883-020-02038-y

**Published:** 2021-01-11

**Authors:** Shouzi Zhang, Yuanyuan Wang, Lixin Liu, Li Zhang, Li Ma, Haiyan Wu, Xuelin He, Mingwei Zhu, Luning Wang, Fan Mei

**Affiliations:** 1 Department of Psychiatry, Beijing Geriatric Hospital, 100095 Beijing, P.R. China; 2 Chinese People’s Liberation Army General Hospital, Beijing, 100853 P.R. China; 3 Institute of Systems Biomedicine, Peking University Health Science Center, Beijing, 100191 P.R. China

**Keywords:** Vascular parkinsonism(VaP), Parkinson's disease(PD), Cerebral small vessel disease (CSVD), Vascular dementia(VaD)

## Abstract

**Background:**

Vascular Parkinsonism(VaP) is defined as parkinsonism resulting from cerebral vascular disease(CVD), with presence of variable motor and non-motor signs that are corroborated by clinical, anatomic or imaging findings of cerebrovascular disease. Overlapping syndromes with mixed pathologies make VaP difficult to distinguish from primary neurodegenerative parkinsonism.To understand the clinical and pathological features of VaP,we report a case of autopsy confirmed vascular Parkinsonism that was clinical misdiagnosed as idiopathic Parkinson’s disease.Clinical features include early mixed symptoms of dementia,behavioral disturbance and parkinsonism that were similar to Dementia with lewy Body(DLB) and Parkinson disease Dementia(PDD).

**Case presentation:**

A 84-year-old man presented progressive parkinsonism with prominent postural instability, gait impairment, pseudobulbar, early cognitive impairment, irritability, hallucination, urinary symptoms and poor responsiveness to dopaminergic drugs. He was clinically diagnosed as Parkinson disease(PD). In the post-mortem study, we examined Aβ and phospho-tau as pathological biomarker for Alzheimer’s disease(AD), α-synucleing in medulla, pons and midbrain for PD and DLB. Hematoxylin and eosin staining in cerebral cortex, cerebellum and brainstem examines vascular pathological changes and microvascular lesion.Neither Lewy bodies in the substantia nigra ,locus ceruleus and cerebrumnor accumulation of Aβ, neurofibrillary tangles were noted. Instead, there were many cerebral infarctions and widespread arteriosclerosis in the brain. The final brain autopsy supported a diagnosis of VaP not PD.

**Conclusions:**

This case of pathologically confirmed VaP misdiagnosed as idiopathic PD suggested that we must be vigilant about the possibility of VaP for patients with parkinsonisms, cognitive impairments, early behavioral and psychological symptoms,imaging performances of cerebral small vessel disease and other vascular damages.

## Background

Parkinsonism secondary to cerebrovascular diseases and systemic disease can mimic neurodegenerative disorders [1]. Parkinsonism of vascular origin defined as Vascular parkinsonism (VaP) accounted for 4.4–12% of all cases of parkinsonism.There are no specific diagnostic criteria of VaP so far, nor accurate incidence and prevalence rates [2]. Pathological evidence of a vascular disease in the absence of typical PD lesions (e.g. Lewy bodies) is the gold standard.VaP is differentiated from degenerative parkinsonism by neuropathological and clinicoanatomic studies.It can be inferred presumably by vascular cause with multiple strokes or vascular event history. An autopsy for VaP would demonstrate vascular pathology derived from ischemic or hemorrhagic strokes involving the Substantia nigra and/or nigrostriatal pathway [3]. However, a clear history of acute neurological deficits or obvious radiological evidence of previous strokes may not be present, while microvascular pathology can be still identified on autopsy.VaP may be confused with other diseases such as Dementia with Lewy Body(DLB) and Parkinson’s disease in clinics. To recognize the clinial and pathological features of VaP, We report the clinical and pathological features of VaP in an elderly patient with progressive extrapyramidal symptoms and cognitive impairment, who was initially misdiagnosed and treated as idiopathic Parkinson’s disease.

## Case presentation

A 68 year old man started complaining of stiffness of his lower limbs, nightmares, and recurrent falls from bed during his sleep since 2002. He was taken to an outpatient department of neurology on the following year. Brain MRI showed intracranial multiple lacunar infarction, leukoencephalopathy, old cerebral hemorrhage in medulla oblongata pons and slightly cerebral atrophy. In consideration of the combination of limbs stiffness and possible rapid-eye-movement(REM) behavioural disorders such as nightmares and falls from bed, Parkinson’s disease (PD) was diagnosed and levodopa/benserazide was prescribed with slight improvement of rigidity.

In 2004, he developed memory loss, irritability, right hand tremor, discontinuous urinary and fecal incontinence. From then on, he took donepezil hydrochloride (10 mg per day) and sedative drugs (Benzodiazepines). Possible visual hallucination, illusion, yelling, progressive amnesia and gait instability were seen in 2005. In 2006, he developed a significant decline in daily living capability and became wheelchair bound because of frequent falls. In May 2007 the patient was admitted to the Psychological department of Beijing Geriatric Hospital. Neurological examination showed rigidity and weakness of the four limbs, mild aphasia, subtle hand tremor.The deep tendon reflex and muscle tonus were abnormal, and bilateral palmomental reflex and Babinski’s sign were not recognized.No other abnormal neurologic signs were evident.

He had a history of hypertension and hyperlipidemia for 20 years and coronary atherosclerotic heart disease for 14 years. There was no history of autoimmune disease, such as thromboembolic vasculitis.. He had 18 years of education and no family history of dementia or other neurologic diseases.

Apolipoprotein E (APOE) genotype was ε3/ε4. Levels for folic acid, vitamin B12 and thyroxine function were normal. Evaluation of neuropsychological rating scales performed in 2004 and 2007 are reported in Table 1.

**Table 1:** Neuropsychological tests of the patient in 2004 and 2007

Cognitive and motor Function	Test	Score (2004)	Score (2007)
Dementia	MMSE	22	9
	MoCA		4
	CDR		3
Language	BNT		5
Parkinsonism estimation	UPDRS		69
BPSD	NPI		32
Activities of dailyliving	Barthel Index	85	20

*MMSE* Mini-Mental State Examination; *MoCA *Montreal cognitive assesment; *CDR *Clinical dementia rating scale; *BNT *the 30-item Chinese version of the Boston Naming Test; *UPDRS *Unified Parkinson disease rating scale; *BPSD *Behavioral and psychological symptoms of dementia; *NPI *neuropsychological inventory.

**Fig. 1:**
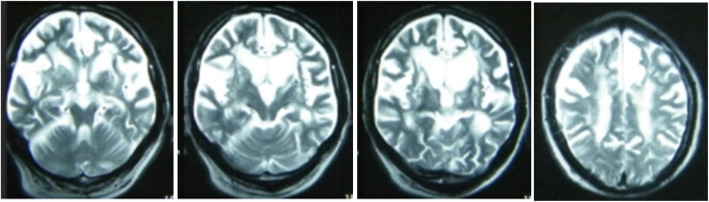
Patient’s brain MRI images Severe atrophy in the bilateral temporal and frontal lobes, mild atrophy in parietal lobes, as well as lacunar infarction in bilateral radiation coronal, basal ganglia, left thalamus and brainstem. Mild white matter hyperintensities (WMHs) can be found on both sides lateral ventricle

3T brain Magnetic Resonance Imaging (MRI, 2007) showed severe atrophy in the bilateral temporal and frontal lobes, mild atrophy in parietal lobes, as well as lacunar infarction in bilateral radiation coronal, basal ganglia, left thalamus and brainstem. Mild white matter hyperintensities (WMHs) can be found on both sides lateral ventricleas.(Fig. 1**)**.

After hospitalization,levodopa/benserazide,Memantine Hydrochloride and rehabilitation treatments were implemented for the patient. He suffered several cerebral vascular diseases on the following years. He developed progressive dysphagia requiring nasogastric tube placement. On March 3, 2018, the patient suddenly developed coma with left limb movement deprived and unequal pupils. A cerebral hemorrhage was suspected and he died 12 hours later.

### Neuropathological examination

**Fig. 2:**
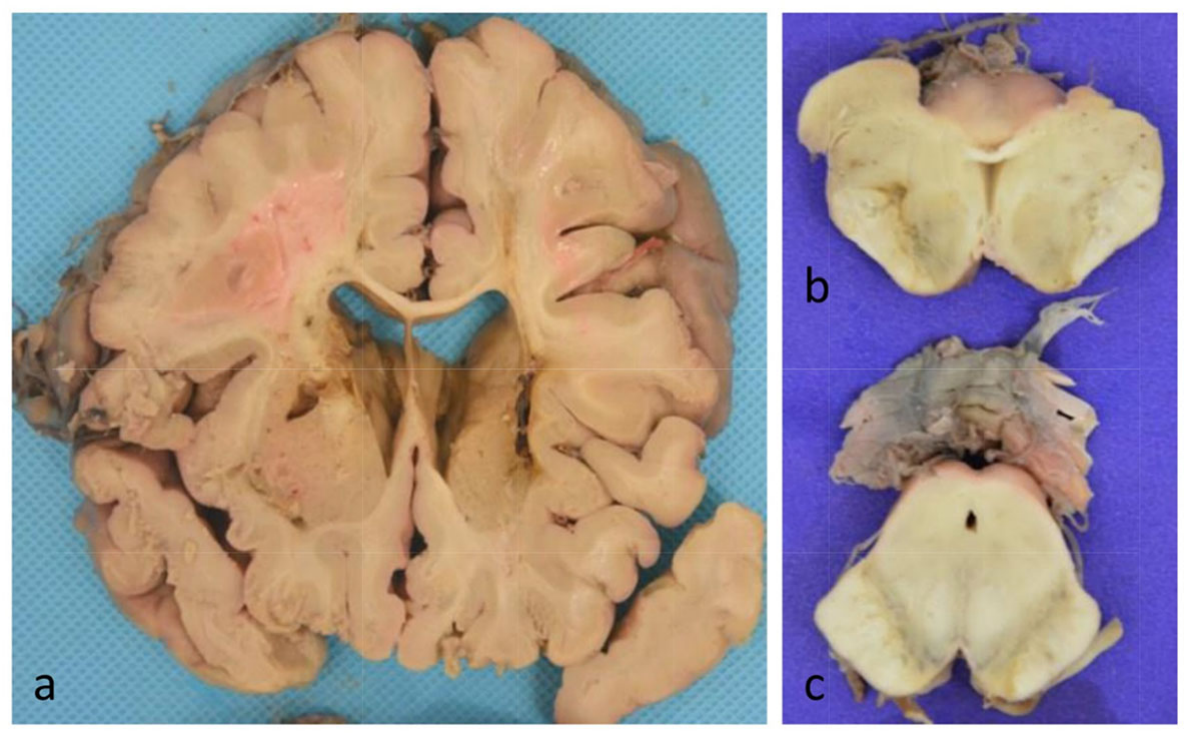
Gross findings: **a** Many micro infarctions in internal capsule, puteman, caudate nucleus and thalamus of both sides. **b** A micro infarction in the midbrain at the level of superior colliculus. **c** Mild depigmentation in the substantia nigra

Postmortem examinations were performed within 24 hours. The brain weighted 1426 g, and brain tissue was fixed in 10% formalin for two weeks. 8-µm thick series sections were mounted, deparaffinized, dehydrated, and stained. Pathologic examination was performed on 13 sections using routine hematoxylin & eosin(HE) (Fig. 2), Luxol fast blue (LFB), modified Gallyas-Braak (GB) silver staining, and immunohistochemical (IHC) staining for Aβ (mouse monoclonal antibody; diluted 1:100; Dako, Glostrup,Denmark), phosphorylated tau (mouse monoclonal clone AT8; diluted 1:500; Thermo Fisher, Rocford,IL,USA), α-synuclein (rabbit polyclonal antibody;diluted 1:1000; Sigma-Aldrich, St.Louis, MO,USA;), TAR DNA binding Protein-43 (TDP-43) (rat monoclonal antibody; pSer409/410; diluted 1:500;Merck Millipore,Temecula,CA, Germany), and P62 (mouse monoclonal clone ;diluted 1:1000; Abnova, Taipei China), according to the National Institute on Aging-Alzheimer’s Association (NIA-AA) guidelines for AD neuropathologic changes (ADNC) [4].

### Neuropathological macroscopic finding

**Fig. 3:**
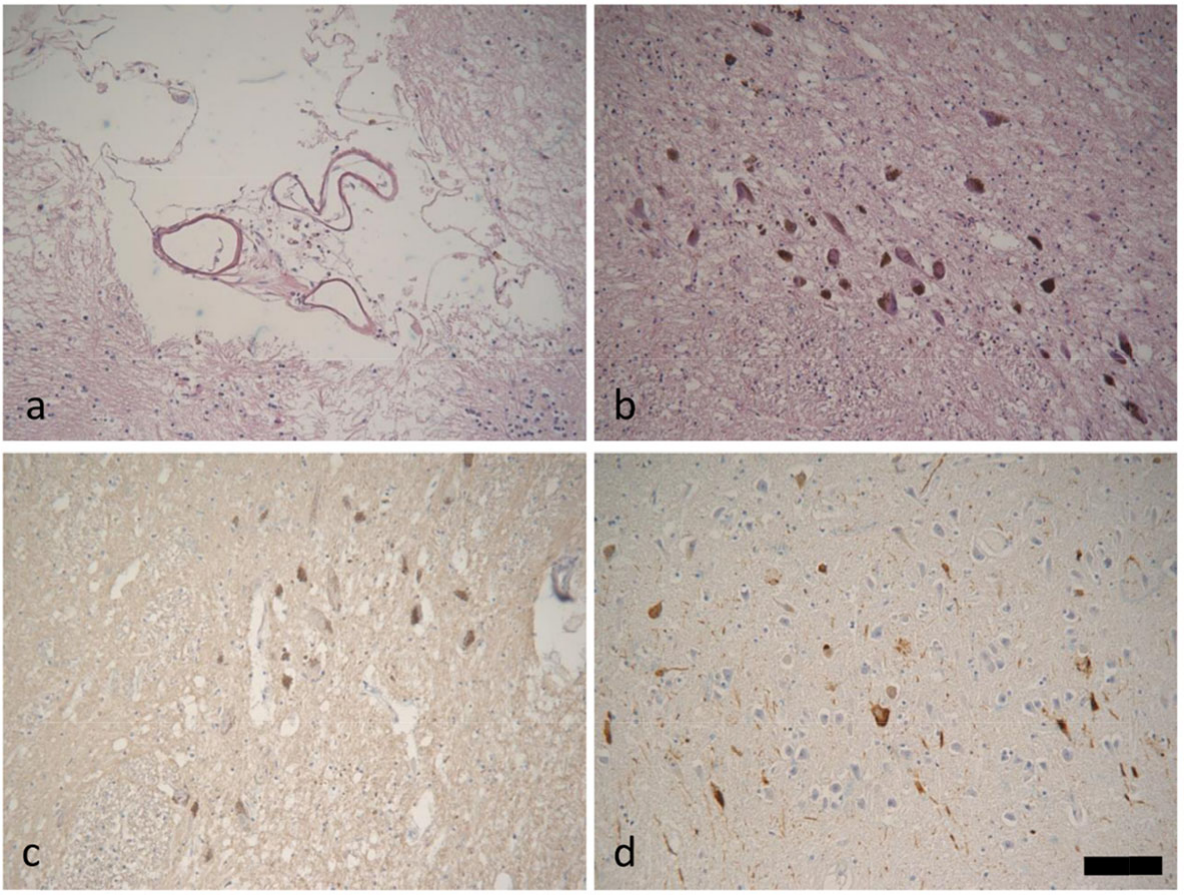
Microscopic findings. **a** Perivascular tissue necrosis with several hemosiderin cells. **b** Slightly depigmentation and no Lewy bodies in the substantia nigra. **c** No Lewy bodies or Lewy threads in the substantia nigra. **d** Neurofibrillary tangles and thresds in the CA1 segment of Hippocampus. HE stain (**a**, **b**), α-synuclein immunostain(c),AT immunostain (**d**). Scale bars:100 μm (**a, b, c, d**)

Severe whole brain edema was observed. There were many cerebral infarctions in the middle cerebral artery. The locus coeruleus was relatively preserved. There was mild midbrain, pons, basal ganglia, thalamus, hemi-ovary center (Figs. 3a and b and 2a). Widespread arteriosclerosis was seen in large, middle and small cerebral vessels, and thrombosis was found in the lumen of the right internal carotid artery and bilateral depigmentation in the substantia nigra (Fig. 3c).

### Neuropathological microscopic findings

Volume shrinkage of neurons were observed in cerebrum, brainstem and cerebellum due to brain edema. Marked Purkinje’s cell loss were found in the cerebellem. We have not found Lewy bodies in the substantia nigra, locus ceruleus and cerebrum by HE staining (Fig. 2b). AT8 and P62 immunoreactive neurofibrillary tangles and threads were seen in the hippocampus, which were also revealed by Gallyas-Braak staining (Fig. 2d). No accumulation of Aβ, α-synuclein or TDP-43 were noted in the brain (Fig. 2c). The Alzheimer’s disease neuropathologic change was A0, B1, C0 (Braak stage I) according to diagnostic criteria made by the National Institute for Aging-Alzheimer Association [3]. There were no astrocytic plaques or tufted astrocytes in the brain or brainstem(data not shown).Based on above fingdings Vascular parkinsonism(VaP) pathologic diagnosis was made.

## Discussion and conclusions

Different movement disorders have been reported after cerebrovascular events, such as parkinsonism, tremor, dystonia, myoclonus, asterixis, stereotypies, akathisia, tics, choreaand freezing of gait.The frequency of cerebravascular abnormal movements has been estimated to vary from 1–4% of all strokes [5]. Vascular Parkinsonism (VaP) is very commonand has been found to be present in about 3–5% of post-mortem studies of patients with parkinsonism [6].The frequency of post-stroke movement disorders is likely to be underestimated. A prospective cohort study reported the incident of VaP over a mean period of 5.2 years in 15 patients (3%) out of 503 patients with cerebral small vessel disease who had no parkinsonism at baseline [7], indicating that pure forms of VaP are relatively rare. A previous study reported 28 pathologically confirmed VaP cases of whom only six were diagnosed as VaP during lifetime and the remainder as either PD or atypical parkinsonism [8]. Thus, VaP is often misdiagnosed as in the case that we reported in this manuscript.

This patient have showed rapid progressed parkinsonisms and cognitive impairment, as well as early behavioral and psychological symptoms. In the neuropathological examination, many cerebral infarctions can be seen in various areas of the brain, especially in the basal ganglia. There were no Lewy bodies, α-synuclein, accumulation of Aβ and proliferation of glial cells. The mild depigmentation in the substantia nigra can be interpreted as a result of aging, being comparable with the findings of aged-matched subjects with no symptoms of PD [9].Thus,A diagnosis of Vascular parkinsonism(VaP) was made and Parkinson’s disease or DLB were excluded. AD neuropathologic change should be ranked based on three Parameters: Aβ plaque score, Braak NFT stage,and neuritic plaque score to obtain an ‘‘ABC score’’. The ABC scores are transformed into one of four levels of AD neuropathologic change: Not, Low, Intermediate or High. In this patient, mild neurofibrillary tangles(NFL) and threads were seen in the hippocampus (Braak stage I), and no accumulation of A β and neuritic plaques. The Alzheimer’s disease neuropathologic change was A0, B1, C0, and not fits the diagnostic standard of AD[4, 10].

An academic working group recommends definition of vascular parkinsonism into three subtype by acute or subacute post-stroke VaP subtype, insidious onset VaPand mixed or overlapping syndromes of idiopathic Parkinson’s disease or other neurodegenerative parkinsonisms and comorbid cerebral vescular disease (CVD) [11]. The acute or subacute post-stroke VaP subtype generally responds to dopaminergic drugs and is typically asymmetric,presenting acute or subacute onset of parkinsonism. Insidious onset VaP subtype is more frequent, presenting progressive parkinsonism with prominent postural instability, gait impairment,corticospinal, pseudobulbar, cerebellar, cognitive and urinary symptoms and tends to be poor responsive to dopaminergic drugs.It would manisfested as a higher-level gait disorder frequently in the clinical spectrum [12]. Mixed or overlapping syndromes of idiopathic Parkinson’s disease and comorbid CVD is diagnosed based on the molecular imaging biomarkers such as dopamine transporter imaging in clinical practice.

In the insidious onset VaP subtype, the cognitive impairment is very common because of an overlap between Vascular Dementia(VaD) and VaP. Both VaP and VaD have similar frequent occurrence of brain atrophy, white matter hyperintensities and lacunes, and represent different aspects of subcortical vascular encephalopathy. The pathological feature of VaP is brain damage caused by vascular factors. The manifestations were ischemia with main lesions in subcortical white matter, basal ganglia, thalamus and midbrain, but hemorrhage was rare [13].The pathological changes of brain tissue in VaP were mainly lacune and white matter lesions with severe oligodendrocyte loss.

Cerebral small vessel disease (CSVD) can be asymptomatic or manifest as lower body parkinsonism.It refers to a series of clinical, imaging and pathological syndromes caused by affected intracerebral arterioles, capillaries and venules [12]. Clinical manifestations include lacunar infarction, cerebral hemorrhage, subcortical white matter lesions,cerebral microhemorrhage and microinfarction [14]. Age, hypertension and hyperhomocysteinemia are recognized risk factors for cerebrovascular disease in many studies. In the early, middle and late stage of cerebral small vessel disease, cognitive dysfunction and emotional changes can be observed to different extent. Depression, gait instability, dysphagia, bladder sphincter dysfunction and decline in daily living ability are very common [12]. Multiple studies have investigated the relationship of cerebral small vessel disease with dementia, particularly microbleeds and WHM are frequently seen in those with cognitive impairment [15, 16]. The association of MRI-based infarcts with an increased risk of dementia was reported in several population-based studies[17, 18].Autopsy has confirmed that this patient had CSVD, coincident with insidious type of VaP. It is speculated that insidious type of VaP may occur due to disruption in the extra-nigrostriatal white or gray matter vascular lesions and connectivity associated with parkinsonism, evena distinct focal nigrostriatal deficit does not occur[19].

Parkinsonism can also be present in patient with and PD, DLB, Progressive Supranuclear Paralysis(PSP), Multiple System Atrophy(MSA) or Corticobasal Degeneration(CBD) that involve α-synucleinopathy,tau or other proteinopathies[20]. The co-occurrence of clinically manifest primary neurodegenerative parkinsonism with vascular disease is common due to mixed pathologies [21]. Similar mixed coincidence of vascular disease and neurodegenerative parkinsonisms can occur in PSP, MSA or CBD [11].Molecular imaging techniques such as dopaminge transporter imaging is helpful to identify more ‘pure’ sub-types of VaP, as well as indicative ofmixed or overlapping syndromes of neurodegenerative parkinsonism and comorbid CVD. SPECT imaging of striatal presynaptic dopamine transporter (DAT) shows a significant decrease in tracer uptake in PD, while VaP was generally normal. SPECT combined with MRI can effectively differentiate Parkinson’s disease from VaP.123I-metaiodobenzylguanidine(MIBG) can be appliedto differentiate VaP from PD, which indicative of function of cardiac sympathetic nerve. Total MIBG uptake was decreased in patients with PD and DLB, but VaP showed normal or mild decrease [22].

## Data Availability

Data sharing is not applicable to this article as no datasets were generated or analysed during the current study.

## Abbreviations

VaP: Vascular Parkinsonism
DLB: Dementia with lewy Body
CVD: Cerebral vascular disease
CSVD: Cerebral small vessel disease
PD: Parkinson’s disease
WMHs: white matter hyperintensities
PSP: Progressive Supranuclear Paralysis
MSA: Multiple System Atrophy
CBD: Corticobasal Degeneration
DAT: Dopamine transporter
